# Do Individual Differences Influence Moment-by-Moment Reports of Emotion Perceived in Music and Speech Prosody?

**DOI:** 10.3389/fnbeh.2018.00184

**Published:** 2018-08-27

**Authors:** Nicola Dibben, Eduardo Coutinho, José A. Vilar, Graciela Estévez-Pérez

**Affiliations:** ^1^Department of Music, University of Sheffield, Sheffield, United Kingdom; ^2^Department of Music, University of Liverpool, Liverpool, United Kingdom; ^3^Department of Mathematics, University of A Coruña, A Coruña, Spain

**Keywords:** emotion, music, prosody, individual differences, continuous, dimensional

## Abstract

Comparison of emotion perception in music and prosody has the potential to contribute to an understanding of their speculated shared evolutionary origin. Previous research suggests shared sensitivity to and processing of music and speech, but less is known about how emotion perception in the auditory domain might be influenced by individual differences. Personality, emotional intelligence, gender, musical training and age exert some influence on discrete, summative judgments of perceived emotion in music and speech stimuli. However, music and speech are temporal phenomena, and little is known about whether individual differences influence moment-by-moment perception of emotion in these domains. A behavioral study collected two main types of data: continuous ratings of perceived emotion while listening to extracts of music and speech, using a computer interface which modeled emotion on two dimensions (arousal and valence), and demographic information including measures of personality (TIPI) and emotional intelligence (TEIQue-SF). Functional analysis of variance on the time series data revealed a small number of statistically significant differences associated with Emotional Stability, Agreeableness, musical training and age. The results indicate that individual differences exert limited influence on continuous judgments of dynamic, naturalistic expressions. We suggest that this reflects a reliance on acoustic cues to emotion in moment-by-moment judgments of perceived emotions and is further evidence of the shared sensitivity to and processing of music and speech.

## Introduction

Speech and music share acoustic attributes which may form a common ‘code’ for emotion communication in the auditory domain (Juslin and Laukka, 2003). Such commonality forms the basis of claims for a shared evolutionary origin for music and spoken communication (Perlovsky, 2010). Music is widely understood as an affective human activity whose auditory properties can play an important role in emotion communication (Gabrielsson and Lindström, 2010), even, to some extent, across cultures (Thompson and Balkwill, 2010). Likewise, vocal prosody (the changes in intonation, timing and stress accompanying speech) is known to communicate emotion across languages and cultures (Scherer et al., 2001). Studies comparing the perception of emotion in music and speech (Juslin and Laukka, 2003; Ilie and Thompson, 2006; Coutinho and Dibben, 2012), and everyday sounds (Weninger et al., 2013), demonstrate sensitivity to shared acoustic features. Evidence suggests that emotional content of auditory stimuli is decoded, at least partially, by a shared processor (Patel, 2013). Acoustic features are unlikely to account for *all* of any emotional response, but they can account for part of that response and are widely assumed to be the factor most likely to exert an effect across individuals regardless of other differences between them. For this reason, research has focused on identifying relationships between the acoustic features and perceived emotion, treating groups as homogenous.

While the evidence for shared sensitivity and processing is growing, less is known about how emotion perception in the auditory domain might be influenced by differences between individuals. Understanding the factors that influence individual differences in the perception of emotion is essential if we are to develop a more nuanced understanding of emotion perception which has ecological validity and practical application (Brattico and Jacobsen, 2009; Vuoskoski and Eerola, 2011). At the inter-cultural level, comparisons of emotion perception in music and speech prosody reveal that psychophysical cues have similar connotations across cultures (Bowling et al., 2012) but that enculturation can influence interpretation of such cues (Thompson and Balkwill, 2010). Intra-cultural studies (primarily of Western listeners to Western art and popular musics) indicate that temporary mood states and more stable traits may influence perception of emotion in music and/or speech prosody, although the evidence is limited and may depend on the model of emotion used as discussed below. For the purposes of this study we investigate those traits which existing evidence suggests may underlie differences between individuals in perceived emotion, namely, personality, emotional intelligence, musical training, gender and age. Each are examined in turn below with the purpose of revealing the extent to which each contributes to perceived emotion, and whether these differences emerge in moment-by-moment (continuous) or summative judgments.

Some of the variation amongst individuals in perception of emotion in the auditory domain can be attributed to personality differences. Personality traits are associated with affective biases in emotion judgments (Rusting, 1998) due to the interaction of personality with attention, motivation and mood (Edgar et al., 2012): there is evidence that Extraversion and Agreeableness predispose people to perceive positive affect in emotional stimuli, whereas Neuroticism (low Emotional Stability) is associated with sensitivity to negative emotional stimuli (Knyazev et al., 2008), and may be instantiated at a neural level (Brück et al., 2011). These affective biases have been found for discrete, summative judgments of emotion in music (Vuoskoski and Eerola, 2011; Taruffi et al., 2017) and in speech prosody (Burton et al., 2013), but it is unknown whether they influence moment-by-moment judgments of emotion.

Another factor which may influence individual variation in the perception of emotion is emotional intelligence, which is associated with improved emotion perception abilities, including in emotional prosody and music. The construct of emotional intelligence, whether conceived either as a trait or an ability (Cherniss, 2010), is based on the idea that the ability to perceive and use emotions differs amongst individuals. Scores on ability-based measures of emotional intelligence have been found to correlate with accuracy of recognition of intended emotion in pieces of classical music (Resnicow et al., 2004), and in speech and melodic analogs of speech (Trimmer and Cuddy, 2008); and trait emotional intelligence has been found to increase with years of musical training (Petrides et al., 2006) suggesting that musical expertise may benefit affective abilities. One might therefore expect ratings of perceived emotion in music and speech to vary with emotional intelligence, but studies have focused on recognition of discrete emotions (Baltes and Miu, 2014) rather than ratings of moment-by-moment perceived emotion. It is therefore unknown whether continuous measures of emotion are influenced by emotional intelligence.

There is also evidence that both ability-based and trait emotional intelligence interact with gender: women score higher on some aspects of emotional intelligence than men (Petrides and Furnham, 2000; Brackett et al., 2004). Indeed, a meta-analysis of sex differences in the ability to recognize discrete emotions in a variety of non-verbal domains indicates that women perform slightly better overall, especially for negative emotions (Thompson and Voyer, 2014). However, the effect size for sex difference is dependent on modality and the authors argue that more data from the auditory domain is required to determine their influence.

Evidence for the effects of musical expertise on emotion perception in music and/or speech prosody is limited and conflicting. Some studies of emotion perception in music suggest musical training has little influence (Bigand and Poulin-Charronnat, 2006; Ramos et al., 2011), whereas others show a correlation between years of musical training and emotion perception in music (Livingstone et al., 2010; Lima and Castro, 2011a). Similarly, some studies of emotion perception in speech prosody show a positive effect of musical training (Thompson et al., 2004; Lima and Castro, 2011b) and argue that non-results from other studies are a consequence of unrepresentative stimuli and insufficient levels of musical training amongst participants; others suggest that emotional intelligence (and an association with musical expertise) rather than musical training is responsible for increased accuracy of emotion recognition in speech (Trimmer and Cuddy, 2008). There are two reasons why musical expertise might influence emotion perception in both music and speech. First, given the strong evidence that emotions arise in large part from features of the stimulus, musical expertise may confer advantages in so far as it results in perception of different, or perhaps more nuanced musical structures. Second, if as suspected, processing of emotions in music and speech engages common mechanisms then musicians, who have had many years of training and exposure, should display enhanced processing of emotion prosody (Magne et al., 2016). Whether such processing advantages might manifest as inter-individual differences in moment-by-moment perception of emotion is unknown.

Lastly, judgments of emotion in non-verbal displays are also influenced by age (Lima et al., 2014). Research into summative judgments of discrete stimuli in a variety of non-musical, non-verbal displays shows that emotion recognition is at its peak in young adults and declines with age (Ruffman et al., 2008). This is the case not only for adults over 60, where cognitive and hearing decline might be expected (Mitchell et al., 2011) but even earlier, from middle age (Paulmann et al., 2008). Some studies of dicrete, summative judgments of emotion recognition have found an age-related bias toward perception of positive emotions, and less sensitivity to negative emotions in both speech and music (Mather and Carstensen, 2005; Laukka and Juslin, 2007; Lima and Castro, 2011a; Vieillard et al., 2012). There is also evidence that older adults discriminate the arousal qualities less than younger adults (Laukka and Juslin, 2007; Vieillard et al., 2012). It has been proposed that this apparent shift toward perception of positive valence with age may be due to a combination of emotion-specific effects of brain aging, and motivational changes over the lifespan (Laukka and Juslin, 2007; Lima and Castro, 2011a), and that changes in sensitivity to arousal may reflect a general reduction of emotional complexity (Vieillard et al., 2012). However, recent evidence adopting continuous measures of emotion with naturalistic, dynamic stimuli rather than summative judgments of discrete emotions suggests this age-related decline may be task-dependent (Sze et al., 2012), and a number of researchers are now turning to more naturalistic tasks and stimuli with the view that this may more accurately reflect the full range of ability.

In sum, there is evidence that summative judgments of perceived emotion in music and speech are influenced by a range of individual differences. However, little is known about how such differences influence moment-by-moment judgments of emotion – an important question given the temporal character of auditory and affective experiences. In particular, continuous judgments might be expected to be minimally susceptible to individual differences which some have argued are more likely to influence the reconstructive process of recall (Barrett, 1997; Schubert, 2010, p. 243). Given the scarcity of the available evidence and the absence of direct comparisons of emotion perception of naturally elicited emotion (as opposed to summative judgments of discrete emotions) in music and speech prosody, the study reported here set out to explore the influence of individual differences in these two auditory domains using continuous measurement of perceived emotion. To our knowledge ours is the first direct comparison of the influence of a wide range of individual differences on emotion perception in music and speech prosody using naturalistic stimuli and continuous, dimensional measures of perceived emotion.

### Aims, Design, Research Questions and Hypotheses

This behavioral study was part of a larger behavioral and computational study of the acoustic cues to emotion in music and vocal prosody (Coutinho and Dibben, 2012). The goal of the current study was to discover whether individual differences influence perception of emotion in music and speech prosody on a moment-by-moment basis. We focus on emotion portrayed, as distinct from the emotion felt by listeners, since the two are not necessarily identical in music nor speech (Gabrielsson, 2002; Ilie and Thompson, 2011). The focus on music and speech together allows us to situate our research and findings within a modality-specific conception of emotion perception.

Participants listened to excerpts of music and speech and rated the perceived emotions. Beyond this, the design of the study differs in important ways from previous research. Typical laboratory studies of emotion perception in music and speech offer excellent experimental control but at the expense of ecological validity. In contrast, we used a task and materials that approximated more closely the dynamic quality of emotion judgments in everyday life. First, the study elicited emotion judgments on unaltered stimuli. Previous research has tended to use relatively short music and speech stimuli specifically designed to represent discrete emotions, rather than using instances of naturally occurring speech or music. Our more naturalistic stimuli allowed investigation of a range of possible acoustic features, affective states, and ‘journeys’ through them. This is important because recent studies using naturalistic, dynamic stimuli in other modalities have found different effects, and advantages for certain groups: older adults perform better in emotion judgments with dynamic, real-world visual stimuli than younger adults (Sze et al., 2012).

Second, we adopted a dimensional rather than discrete model of emotion: emotional responses to the stimuli were recorded along two axes (valence and arousal), which represent specific emotions as points within a two-dimensional space. A two-dimensional model was adopted in this study because it facilitates representation of a wide range of mild and full-blown emotions, allows direct comparison of music and speech emotional ratings, is reliable and economical, and can be implemented for the collection, analysis and modeling of continuous data (Eerola and Vuoskoski, 2011; Vuoskoski and Eerola, 2011).

Third, we tested for effects of individual differences based on continuous rather than summative ratings of emotion. Continuous measures are sensitive to the temporal character of music and speech, can track an emotional journey through various affective qualities, and have good test–retest reliability (Schubert, 2013). Indeed, continuous measures are beginning to be used in emotion research in other modalities, specifically in facial expression research (Edgar et al., 2012; Sze et al., 2012), where it has contradicted some of the findings from summative judgments. By its nature, a summative evaluation is a post-hoc, holistic judgment and it is unclear how it reflects the temporal characteristics of an unfolding emotional experience. Summative evaluations do not correspond to an arithmetic mean of the listener’s experience on a particular dimension, particularly when that stimuli varies on that dimension over its duration. Instead summative evaluations appear to be influenced by a complex interplay of temporal and qualitative aspects of the experience (Brittin and Sheldon, 1995; Brittin and Duke, 1997; Duke and Colprit, 2001; Schubert, 2010), and are potentially mediated by the effects of personality traits on the constructive process of recall (Barrett, 1997). By contrast, using a continuous measure allowed us to investigate the effects of individual differences on perceived emotion over the time course of listening – something as yet unexplored.

We hypothesized that continuous, dimensional judgments of emotion perception would be influenced by individual differences, since those found in discrete emotion judgments could be expected to exert some influence on continuous judgments. However, given the high predictive value of acoustic features in moment-to-moment models of emotion perception (Coutinho and Dibben, 2012) we anticipated that inter-individual variation in this context would be minimal. It should be noted that forming hypotheses about the influence of individual differences on continuous, dimensional judgments of emotion from the existing evidence is not straightforward in this case: authors of studies investigating recognition of summative, discrete emotions have tended to frame their results in terms of the ‘accuracy’ of emotion perception, judged against the ‘intended’ emotion, whereas we ask whether and how groups differ one from another. Our hypotheses are therefore framed as predictions regarding the presence (or absence) of differences between groups.

Based on the existing evidence reviewed above, we formed three hypotheses. Hypothesis 1: We predicted that Emotional Stability and Agreeableness and Extraversion would influence ratings, with those scoring high on Neuroticism more likely to perceive negative, low arousal emotion, and those high on Extraversion and Agreeableness to perceive less negative, low arousal emotion. Hypothesis 2: We anticipated that musical expertise (conceptualized as level of musical training) would result in differences in perceived emotion in music, on the basis that expert listeners might attend to different musical structures. Hypothesis 3: We expected age-related bias away from perception of negative emotions by older participants. Emotional intelligence, and its interaction with sex, is associated with accuracy of emotion recognition, but there is no evidence that it is associated with particular affective biases. Therefore, we did not expect judgments of perceived emotion to be influenced by emotional intelligence or gender.

One feature this summary highlights is that some predicted effects of individual differences are affect-specific, that is, they apply to a greater or lesser extent and in different ways according to the particular discrete emotion, or portion of the affective space, under scrutiny. This has important implications for our focus on continuous judgments of stimuli representing a range of emotions within each example (as opposed to summative judgments on stimuli representing discrete emotions). Specifically, naturally occurring music and speech stimuli, as used here, undergo change in their emotional profile during the course of their temporal unfolding, and, as a consequence of this, any effects of individual differences are likely to impact on parts of the affective space more than others, and therefore during portions of the rating of any particular stimuli rather than uniformly throughout its duration. We therefore predicted that individual differences would have most impact on ratings during specific regions of stimuli in line with the specific affect-dependent predictions above.

In sum, our study was designed to investigate the influence of individual differences on continuous, dimensional evaluations of emotion in naturalistic stimuli, with the aim of providing insight into the moment-by-moment experience of emotion.

## Materials and Methods

### Participants

Sixty volunteers participated in the experiment. Two participants were excluded from the analysis due to measurement errors, and a further six were removed whose native language was not English. The purpose of the latter selection was to minimize variability in responses to the language samples. The final dataset used for the analysis consists of 52 participants (mean age = 32, *SD* = 13, range = 18–62 years; 26 females and 26 males). Participants had a range of musical training (<1 year formal music training = 15; 1–10 years music training = 20; >10 years formal training = 17; median = 5–10 years of formal music training), and reported enjoying film music (the mean rating was 3.9 on a 5-point Likert item where 1 corresponds to “I hate film music” and 5 corresponds to “I love film music”). All but one reported being exposed to film music at least “occasionally.”

### Materials

The stimulus materials consisted of eight extracts of music, and nine samples of speech. In order to achieve comparable ecological validity, the music stimuli were excerpted from late twentieth century Hollywood film soundtracks and the speech stimuli were excerpted from publicly available film, dramatic performances, poetry recitations and documentary interviews; these genres of music and speech are widely experienced and intended to communicate emotion. Moreover, the speech stimuli were chosen to include examples of both spontaneous and enacted speech due to potential differences in the way these represent emotion. The music and speech excerpts were selected from a larger set of twenty speech and twenty music pieces, and comprised a wide variety of instrumentation (in the case of music) and emotional variety and range. Sub-selection was made from this set via pre-testing with 15 student and staff participants from the University of Sheffield using a paper-based self-report two-dimensional affect space. Selection of the final set of stimuli was determined by three criteria: highest consistency of emotion rating for the individual excerpts among respondents; widest coverage of emotions conceptualized as a two-dimensional emotional space (2DES) both by individual excerpts over their time course (averaged across participants), and by the set as a whole; a diversity of psychoacoustic dimensions represented by the set as a whole (e.g., instrumentation, loudness, tempo). The “coverage” of the two-dimensional affect space provided by the stimuli improves the generalizability of the findings to a wide range of emotion states. The stimuli were up to two and half minutes in length, in order to allow measurement of dynamic changes in affective experience, and to keep the total experiment less than 30 min in duration. Although a dimensional reduction from ratings performed on a large set of stimuli is often ideal in stimuli selection (Eerola and Vuoskoski, 2011), it was not feasible to use such method to discriminate stimuli according to the excerpt selection criteria needed for this experiment as described above.

The music used is shown in **Table 1**. The multidimensional emotion qualities communicated by each piece as determined by pre-testing is indicated by the labels Q_1_ to Q_4_, which represent the four main areas resulting from a division of the 2DES arousal/valence diagram into quadrants: Quadrant 1 (Q_1_) – positive arousal and positive valence, Quadrant 2 (Q_2_) – positive arousal and negative valence, Quadrant 3 (Q_3_) – negative arousal and negative valence, and Quadrant 4 (Q_4_) – negative arousal and positive valence.

**Table 1:** Pieces of music used in the empirical study.

ID	*Piece*	Duration	Expected quadrant
1	*(Bram Stoker’s) Dracula*, Vampire Hunters	2:00	Q2
2	*Bride of Frankenstein*, Main Title	1:24	Q1 Q2 Q3 Q4
3	*Guns for San Sebastian*	1:54	Q1 Q3
4	*Hellraiser*, Main Title	1:50	Q2 Q3
5	*Krull*, Love Theme	2:10	Q1 Q4
6	*Minority Report*, Main Theme	1:48	Q1 Q4
7	*The Quiet Earth*, Finale	1:48	Q2 Q3
8	*The Searchers*, Suite	1:29	Q1 Q2 Q4


*The extracts are numbered consecutively, so as to serve as aliases for reference in this article. For each extract we give the title, its duration, and the 2DES quadrant corresponding to the emotional response we expect the extract to elicit in listeners based on pre-testing. Quadrant 1 (Q_*1*_) – positive arousal and positive valence, Quadrant 2 (Q_*2*_) – positive arousal and negative valence, Quadrant 3 (Q_*3*_) – negative arousal and negative valence, and Quadrant 4 (Q_*4*_) – negative arousal and positive valence. All sound stimuli sources are listed in the **Discography***.

The speech samples were chosen to be all from the same language, and in a language not understood by participants. This was necessary in order to avoid any confounds due to the necessarily different semantic content of ecological speech samples. German was selected due to evidence in previous research that native English speakers not conversant in German are able to decode the emotional nuances of German prosody (Scherer et al., 2001; Thompson and Balkwill, 2006). The speech samples used are shown in **Table 2**. As above, the emotions communicated by each excerpt, as determined by the pre-test, is indicated by the labels Q_1_ to Q_4_.

**Table 2:** Speech samples used in the experiment.

ID	Sample	Duration	Expected quadrant
1	Sketch: “The doctrine of the four temperaments: Mr. Sanguinix.”	0:45	Q1
2	Interview: Charlotte Roche “Woman secrets.”	2:36	Q1 Q4
3	Speech: Howard Beale (actor Peter Finch) delivering his “mad as hell” speech from the film *Network*.	1:39	Q2
4	Sketch: “The doctrine of the four temperaments: Mr. Cholerix.”	0:58	Q2
5	Interview: Jenny Spritzer “Ich Bin Ein Wunder/I Am A Miracle.”	1:16	Q2 Q3
6	Poetry: “Orphische Bucht” (Orphic Bay). Poem by Erich Arendt (recited by an unknown female).	1:40	Q3 Q4
7	Speech: Albert Jerska (actor Volkmar Kleinert) speaking to Georg Dreyman (actor Sebastian Koch) in the film *The Lives of Others/Das Leben Der Anderen*.	1:03	Q3
8	Interview: Njeri Weth “A voice that touches.”	1:28	Q1 Q4
9	Interview: Edda Raspé “Three things that make me happy.”	1:46	Q3 Q4


*The excerpts are numbered consecutively, so as to serve as aliases for reference in this article. For each excerpt we indicate the source, its duration, and the 2DES quadrant corresponding to the emotional response we expect it to elicit in listeners, based on pre-testing. Quadrant 1 (Q_*1*_) – positive arousal and positive valence, Quadrant 2 (Q_*2*_) – positive arousal and negative valence, Quadrant 3 (Q_*3*_) – negative arousal and negative valence, and Quadrant 4 (Q_*4*_) – negative arousal and positive valence. All sound stimuli sources are listed in the **Discography***.

To gather data on mood state during the experiment participants completed a Valence-Arousal mood scale (Aarts et al., 2007). This comprises three items measuring valence (*bad–good*, *sad–happy*, and *displeased–pleased)* and three items measuring arousal (*calm–excited*, *tired–energetic*, and *sedate–aroused*), which are responded to on a semantic differential scale (-3 = > 0 = > 3). This scale has been shown to be a reliable measure of two separate variables (Aarts et al., 2007) which can be related to the two dimensions of valence and arousal.

Participants also completed a questionnaire collecting information on demographics. This included 5-point Likert items for musical training, musical exposure, and musical enjoyment. Two further questionnaires gathered information on Personality and Emotional Intelligence. Personality was assessed using the Ten-Item Personality Inventory (TIPI), which is a brief measure of the Big-Five personality dimensions (Gosling et al., 2003), which is the most widely used and researched model of personality and has been used in comparable studies investigating effects of individual differences on emotion perception (e.g., Laukka and Juslin, 2007). This empirically derived framework proposes that individual differences in personality are accounted for by five broad dimensions which subsume within them other hierarchically organized clusters of characteristics (Costa and McCrae, 1992): Agreeableness (A), Conscientiousness (C), Emotional Stability (ES) (or “Neuroticism”), Extraversion (E), and Openness to Experience (O). Emotional Intelligence was measured using the 30-item Trait Emotional Intelligence Questionnaire-Short Form (TEIQue-SF: Petrides and Furnham, 2006). We adopt a trait based measure (of social and emotional competence) here because it has been shown to account for variance beyond that of the Big Five (Petrides et al., 2004). In both cases participants respond to the items using 7-point scales.

### Equipment

A continuous response method was used to obtain fine-grained temporal variations in reported emotional experience collected using software constructed by the second author, which consists of a computer representation of a two-dimensional emotional space (2DES). The two axes are labeled “low valence” and “high valence” at the left and right horizontal extremes respectively, and “low arousal” and “high arousal” at the bottom and top vertical extremes. This interface allows participants to report changes in their emotional state at any moment, instead of doing so only at the end of the piece, and has previously been used successfully in studies of emotional responses to music (e.g., Schubert, 2004; Grewe et al., 2007a,b; Coutinho and Cangelosi, 2011). The output from this self-report method is a time series depicting the dynamics of participants’ ratings of emotion at every moment in the music. Physiological data (blood volume pulse, electrocardiography, skin conductance, and respiration rate) was collected using the ProComp5 Infiniti encoder (Thought Technology Ltd.).

### Procedure

Each participant sat comfortably in a chair inside a quiet room. The goal of the experiment was explained through written instructions that described the quantification of emotion and the self-report framework to be used during the listening task. Participants reported the emotion they perceived by operating a mouse to navigate a computer representation of a two-dimensional emotional space (2DES). Physiological data (blood volume pulse, electrocardiography, skin conductance, and respiration rate) was collected using the ProComp5 Infiniti encoder: participants had sensors attached to their left-hand (if right handed – if not, sensors were attached to the left hand), their chest, and wore a strap around the chest. The physiological data is not reported here since it pertains to a related but separate study.

Each participant was given the opportunity to practice reporting perceived emotion using the self-report framework using ten pictures taken from the International Affective Picture System manual (Lang et al., 2005). The selected pictures represented emotions covering all four quadrants of the 2DES (two per quadrant), and the neutral affective state (center of the axes). The pictures were shown in a non-randomized order, in order to avoid starting or finishing the picture slideshow with a scene of violence. Each picture was shown for 30 s, with a 10 s delay between presentations. The only aim of this exercise was to familiarize participants with the use of the self-report framework.

After the practice period, participants were asked about their understanding of the experiment, and whether they felt comfortable in reporting the intended affective states with the software provided. Participants were then reminded to rate the emotions expressed by the music and speech stimuli, and not the ones felt. When the participant was ready, the main experiment started and the first stimulus was played. The stimuli were presented in a randomized order, with a break of 75 s between each excerpt (unless the participant needed more time). Each experimental session lasted for about 60 min, including debrief, preparation and training periods.

### Data Processing

The datasets from this study are available from https://zenodo.org/record/345944#.Wvv1EMvw9EY, as collection MP_DB3_.

### Self-Report Variables

The arousal and valence reported by each participant was recorded from the mouse movements. These values were normalized to a continuous scale ranging from -1 to 1, with 0 as neutral. The central tendency of the individual values of arousal and valence was estimated by calculating the arithmetic mean across all participants, on a second by second basis, for each sound stimulus.

### Emotional Intelligence, Personality, Musical Training and Age

Values obtained from participants do not differ significantly from the comparable TIPI “White” ethnicity population norms (as reported in Gosling et al., 2003). Norms for TEIQue are not available for comparison. In the case of personality and Emotional Intelligence measures participants were separated into subgroups according to whether they were above or below the respective mean (indicated by the sign of the *z*-score). This resulted in two groups categorized by Emotional Intelligence (High: *n* = 28; Low: *n* = 24), and two groups for each of five personality constructs: Extroversion (High: *n* = 26; Low: *n* = 26), Agreeableness (High: *n* = 28; Low: *n* = 24), Conscientiousness (High: *n* = 38; Low = 14), Emotional Stability (High: *n* = 27; Low: *n* = 25), Openness to New Experiences (High: *n* = 32; Low: *n* = 20). Given evidence that any effect of musical training on emotion perception may only emerge with extensive musical experience (Lima and Castro, 2011a) we categorized participants as “trained” musicians if they had ten or more years of musical training, and “untrained” if they had less than one year of musical training (<1 year formal music training = 15; >10 years formal training = 17). In the case of age, we categorized participants using a split at > 40 years of age (Young: *n* = 40; Old: *n* = 12), based on previous research which identified a decline in emotional prosody perception from this age (Paulmann et al., 2008).

### Mood State

The mean of each of the 3 items related to arousal and valence was calculated to produce two separate measures: arousal-mood and valence-mood.

### Stimuli

The analyses examined associations for each individual stimulus separately, that is, piece by piece, and speech sample by speech sample, rather than together (i.e., a single ‘joined’ music stimulus, and a speech stimulus). Stimuli are analyzed separately because we hypothesized that individual differences would emerge for stimuli dependent on the quadrant, and journey through, the two-dimensional affect space.

## Results

### Stimuli

**FIGURE 1:**
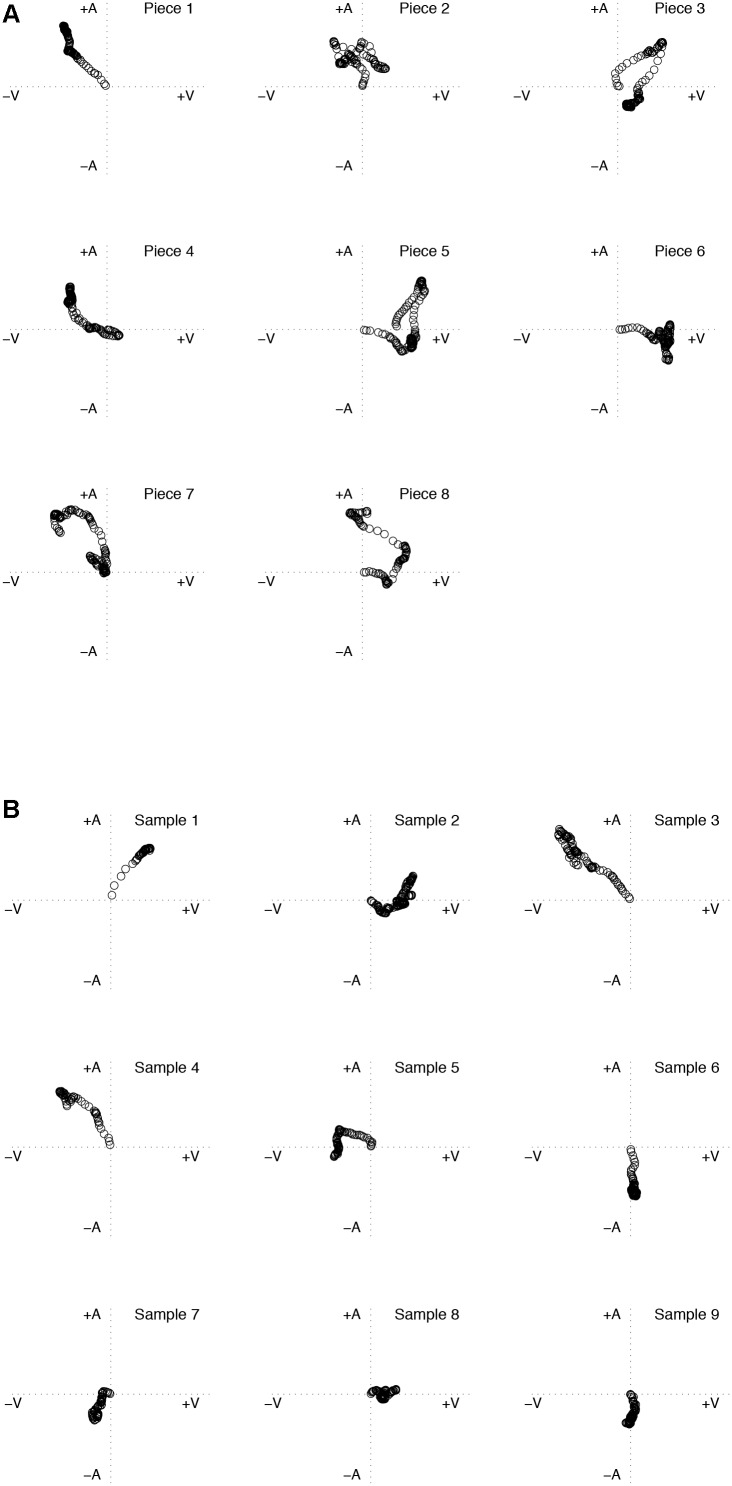
Plot showing the second-by-second values of the self-reported emotional arousal and valence averaged across all participants at each time point for each music piece **(A)** and speech sample **(B)**. Each pair of values is represented by their corresponding location in the 2DES.

We tested the representativeness of the stimuli in terms of their perceived location and movement within the 2DES affect space. The music stimuli elicited responses in the predicted quadrants of the 2DES (**Table 1** shows predicted quadrants, **Figure 1A** shows elicited quandrants). As intended, responses for individual pieces showed changes during the time course of the stimuli and responses across the set of stimuli covered all four quadrants of the 2DES (see **Figure 1A**). Ratings are slightly skewed toward the higher half of the arousal dimension in the 2DES, which may reflect the particular stimuli chosen. Similarly, the speech extracts elicited responses in the predicted quadrants of the 2DES (**Table 2** shows the predicted quandrants). Responses for individual pieces showed changes during the time course of the stimuli and responses across the set of extracts covered all four quadrants of the 2DES (see **Figure 1B**), with individual participants using the full range of the 2DES. These results confirm that the stimuli represent a variety of emotion states and journeys through the two-dimensional emotion space. A detailed analysis of the relationship between psychoacoustic features and judgments of emotion is reported elsewhere (Coutinho and Dibben, 2012).

### Reliability of the Means

The internal consistency of participants’ ratings of emotions across all stimuli (as measured by Cronbach’s Alpha) was high for both reported arousal (0.95) and valence (0.80), in keeping with comparable studies (Schubert, 2013). Cronbach’s Alpha scores for each individual piece and speech sample showed very high consistency (all > 0.80), with the exception of the arousal score for speech sample 8 (0.65) and the valence scores for speech samples 6 (-0.73) and 9 (0.15) (**Table 3**). Therefore results for speech samples 6, 8, and 9 on these dimensions, are omitted from further analysis.

**Table 3:** Cronbach’s Alpha indicating the reliability of participants’ ratings of arousal and valence for each stimulus using the individual time series.

Stimulus ID	Arousal	Valence
Piece 1	0.98	0.94
Piece 2	0.94	0.97
Piece 3	0.99	0.89
Piece 4	0.98	0.98
Piece 5	0.99	0.95
Piece 6	0.93	0.96
Piece 7	0.99	0.98
Piece 8	0.99	0.97
Sample 1	0.99	0.97
Sample 2	0.96	0.95
Sample 3	0.99	0.99
Sample 4	0.99	0.99
Sample 5	0.96	0.97
Sample 6	0.97	0.73
Sample 7	0.96	0.78
Sample 8	0.65	0.89
Sample 9	0.95	0.15


The internal consistency of the personality and emotional intelligence scales was similar to the relevant reference norms. The TIPI showed Cronbach’s Alpha scores consistent with reference values: Extroversion α = 0.64 (reference α = 0.68); Agreeableness α = 0.21 (reference α = 0.40); Conscientiousness α = 0.42 (reference α = 0.50); Emotional Stability α = 0.58 (reference α = 0.73); Openness α = 0.41 (reference α = 0.45). The TEIQue-SF was similarly consistent with reference norms: EI α = 0.81 (reference α = 0.88).

### Independence of the Means

In order to check whether gender, musical training, personality, trait emotional intelligence and age influenced participants’ ratings of valence and arousal, functional analyses of variance were calculated on the time series data. The continuous recorded observations were downsampled and therefore have the nature of functional data by presenting a number of grid points (ranging from 45 to 156) substantially larger than the number of participants (52). Moreover, we are specifically interested in comparing the temporal dynamics of the emotion measures, and therefore the use of models that take into account the continuity of the complete trajectories through time seems to be an appropriate approach here. Note that a standard multivariate anova method (MANOVA) would be strongly affected by dimensionality; namely, when analyzing data in high-dimensional spaces the amount of data needed grows exponentially with the increasing number of dimensions, and the sparsity of data within the high-dimensional space prevents the grouping necessary to commonly used organizational strategies. A dimension reduction technique such as a principal component analysis would be required, but in that case, we face the difficulty of interpreting the selected principal components. In short, the benefits of using infinite-dimensional techniques are very clear in our study.

We applied the functional ANOVA test proposed by Estévez-Pérez and Vilar (2013), which consists of the following steps. First, the raw data are smoothed using local polynomial regression methods (Fan and Gijbels, 1996) and then the estimated curves under each experimental condition are averaged to approximate the corresponding mean functions. These mean curves are then used to construct a test statistic of Cramér-von Mises type whose distribution under the null hypothesis of equality of mean functions is approximated by functional bootstrap. The resulting test is purely functional and non-parametric and presents the additional advantage of being valid to deal with heteroscedastic data. Our analyses involve multiple tests, therefore correction was applied using the Benjamini and Hochberg (BH) procedure (Benjamini and Hochberg, 1995). BH correction is based on controlling the false discovery rate (FDR), that is the expected value of the proportion of Type I errors among the rejected hypotheses. FDR-based methods are typically more powerful than those based on controlling the family wise error rate (FWER), such as Bonferroni correction. Given a set of n simultaneous tests, the FWER is the probability of having at least one Type I error through the several hypotheses under consideration. The Bonferroni correction is based on the idea of maintaining the FWER by testing each individual hypothesis at a statistical significance level of 1 – n times what it would be if only one hypothesis was tested. Bonferroni procedure is very conservative, i.e., it has a remarkable lack of power, which is greatly reduced when the number of hypothesis increases. In our case, n takes the value 8 for each stimulus (music or speech). The results below therefore use the BH correction procedure.

**FIGURE 2:**
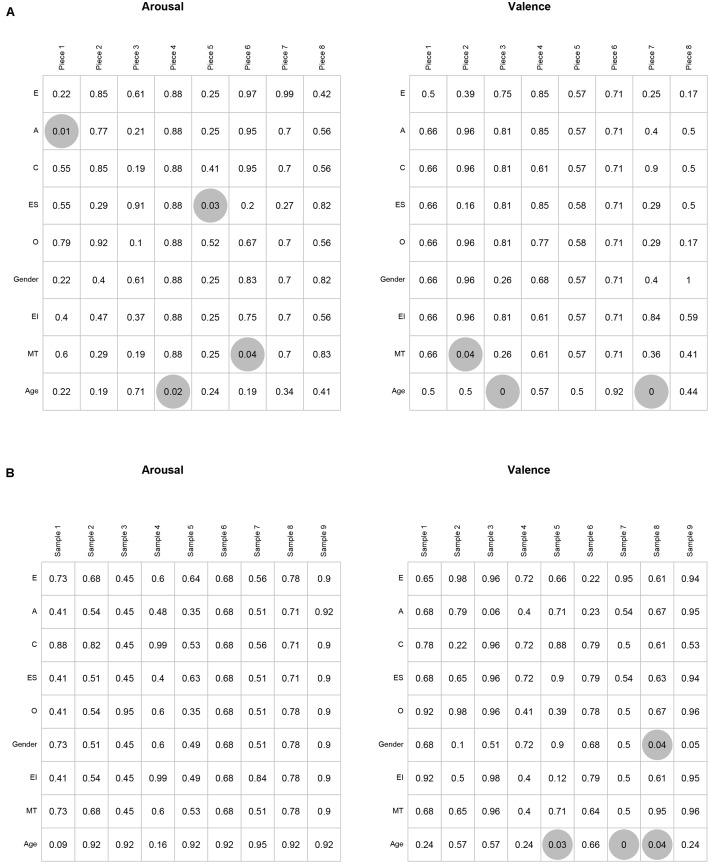
Results of functional ANOVAs on ratings of arousal and valence of music **(A)** and speech **(B)** according to personality (E, Extroversion; A, Agreeableness; C, Conscientiousness; ES, Emotional Stability; O, Openness to new experiences), gender, emotional intelligence (EI), musical training (MT), and age. Gray circles represent significance at *p* < 0.05, with BH correction applied.

Overall there were very few significant differences between groups: in less than 4% of the 306 tests ([8 pieces × 2 variables × 9 groups] + [9 speech samples × 2 variables × 9 groups]) were there statistically significant differences (*p* < 0.05). There were seven significant differences for the music stimuli and four for speech (**Figure 2**), which are dealt with in turn below.

First, a significant effect of personality was found for perceived emotion in two pieces of music, but for arousal rather than valence, contrary to expectations. High and low Emotional Stability groups differed significantly in the arousal reported for music piece 5 (a piece whose ratings lie in the positive, high-arousal quadrant of the emotion space): the group of subjects ranking lower in Emotional Stability (higher Neuroticism) reported higher levels of arousal (**Supplementary Figure 1A**). In addition, groups differing according to high and low Agreeableness reported significantly different arousal for Piece 1 (negative valence, high-arousal): those higher in Agreeableness reported higher arousal (**Supplementary Figure 1B**). Given these were the only significant results for effects of personality on emotion judgments, and Cronbach’s Alpha for Agreeableness was relatively low we do not pursue them further here.

Second, musical training did not exert a large influence on ratings of emotion: the only significant differences are for one dimension for two of the nine stimuli, and, in those two Pieces, the contours of the emotion judgments provided by the two groups are extremely similar (**Supplementary Figure 1C**). This is congruent with previous evidence for a high level of agreement between trained and untrained listeners (Bigand and Poulin-Charronnat, 2006). Given that previous studies suggest there may be enhanced recognition of emotions in music and speech for trained musicians we would expect a difference to emerge in continuous response measures, perhaps manifested as greater sensitivity to musical structures. “Trained” musicians reported significantly higher arousal for Piece 6 than did “untrained,” and significantly lower valence for Piece 2. In the case of Piece 6 (*Minority Report*) the slow tempo and legato phrasing might suggest low arousal, but harmonic suspensions and contrapuntal texture suggest higher arousal. Based on previous work attesting to the role of musical structures in emotion perception (Gabrielsson and Lindström, 2010) and the more fine-grained information available to expert listeners in some cases (Spitzer and Coutinho, 2014) we speculate that the different groups are attending to different aspects of the musical structure. In the case of Piece 2, it is more difficult to attribute the disparity to differences in musical structures. Previous studies elicited single emotion judgments on whole extracts rather than continuous measures; by contrast, our results reveal the nuanced differences that may arise in the time course of listening and responding affectively.

Third, age influenced judgment of emotion in both music and speech. Valence was significantly different as judged by younger and older adults for pieces 3 (*p* = 0.000) and 7 (*p* = 0.002): in both cases the older age group reported more positive valence (**Supplementary Figure 1D**). Arousal was also influenced by age in the case of Piece 4 (*p* = 0.024), which older participants reported as being of lower arousal than younger participants (**Supplementary Figure 1E**). Age was one of only two factors to emerge as significant in the ratings of the speech samples: there was a significant difference between younger and older adults regarding reported valence for speech samples 5 (*p* = 0.027), 7 (*p* < 0.001) and 8 (*p* = 0.044) (**Supplementary Figure 1F**). In each case, the older age group reported more positive valence than the younger age group, which is in keeping with results from discrete, summative judgments of emotion. The other difference which influenced speech perception was gender: men judged the valence of speech sample 8 to be significantly more positive than did the women (**Supplementary Figure 1G**).

Lastly we investigated whether there were individual differences in sensitivity to mood induction as a consequence of listening to the music and speech stimuli. Ratings of mood state (arousal and valence) taken before and after the presentation of the music and speech stimuli were converted to difference scores. Multivariate analyses of variance were performed to compare change of reported mood after music and speech stimuli on each of the two mood measures. Using Pillai’s Trace, there was no significant effect of Emotional Intelligence [*V* = 0.11, *F*(2,47) = 2.9, *p* > 0.05] nor gender [*V* = 0.05, *F*(2,47) = 1.23, *p* > 0.05], nor any interaction between them [*V* = 0.03, *F*(2,47) = 0.63, *p* > 0.05] on mood arousal or valence after presentation of music and speech stimuli. Nor were there any effects of personality or age on changes in the valence and arousal of reported mood state after presentation of music and speech stimuli.

## Discussion

This behavioral study tested whether individual differences influence the perception of emotion in music and speech prosody. Unlike other studies, participants continuously updated their ratings of emotion as the experience unfolded, and did so on dynamic, naturalistic stimuli. Very few significant effects of individual differences were found for the perception of emotion in music, and even fewer for speech prosody. This is contrary to studies using summative judgments and provides the first evidence from both music and speech that recall and moment-by-moment reports result in different characterisations of the experience – something previously only documented for music, and other domains of experience (Schubert, 2010).

An influence of personality (Hypothesis 1) (Agreeableness and Emotional Stability) on judgments of emotion was only found for two of the five music stimuli, and was only partially consistent with results from summative, discrete judgments of emotion which showed affective biases associated with personality (Vuoskoski and Eerola, 2011). In the few cases that emerged, lower levels of Emotional Stability and higher Agreeableness were each associated with high perceived arousal rather than with biases in perceived valence. Musical training (Hypothesis 2) was associated with divergence between judgments of emotion in music in a few instances in our study. This could lend support to the idea that musical expertise results in perception of different, or perhaps more nuanced musical structures, although there is currently insufficient evidence to offer more than speculation on this point. Age (Hypothesis 3) was significantly associated with perceived emotion for both music and speech stimuli: as hypothesized, increasing age was associated with a positive bias in perceived valence. We did not expect to find gender differences in perception of emotion across speech and music and found this to be the case, with only one significant difference on a single speech stimulus). As anticipated, there were no differences associated with emotional intelligence.

The finding that individual differences exert limited influence on moment-by-moment emotion judgments of music and speech is particularly noteworthy given that this is the first time, to our knowledge, that they have been investigated together using continuous measurements. A strength of our findings is that they arise from ecological rather than artificial stimulus materials, and are based upon continuous ratings of emotion, thus capturing the dynamic character of emotional experience with music and speech with mild as well as nuanced emotional states. Moreover, this is the first time to our knowledge that functional analyses have been applied to time series data of this sort. This method enables further investigation of group differences in emotion perception since it is able to capture the temporal dynamics of musical experience (Schubert, 2010).

The limited influence of individual differences on emotion perception in our study may reflect a fundamental difference between the way in which they impact on summative and continuous measures of emotion. This would need to be the subject of a future direct comparison. It may be that continuous measures facilitate a focus on musical structures on a moment-to-moment basis and are therefore strongly influenced by the acoustic features of the music, which, in principle, communicate the same information to all listeners and reduce the range of associations and sources of emotions called upon. Evidence for the high predictive value of acoustic features in music and speech is reported in Coutinho and Dibben (2012). By contrast, summative evaluations represent more complex assessments: a summative evaluation is a best guess approximation to the entire emotional journey, which is particularly problematic in cases where the music expresses a range of emotions during its time course (Brittin and Duke, 1997; Eerola and Vuoskoski, 2011). Notably, averaging continuous responses does not give the same result as a single summative evaluation (Brittin and Sheldon, 1995; Brittin and Duke, 1997), indicating the complexity of summative judgments, and their openness to other influences such as mood state and personality (Vuoskoski and Eerola, 2011). This possible explanation for the difference between the effects of individual differences on continuous responses as measured here, and on summative responses, as previously documented, should be explored through direct comparison of summative and continuous, and dimensional and discrete models in future research. A further important step is to use a variety of implicit and explicit tasks to determine the extent to which listeners are focused on musical structure in these two kinds of judgments.

Three potential limitations of the current study should be considered. First, dichotomization of the individual difference variables is a common technique, which allows comparison with other studies, but it removes potentially informative variance in the predictor variables. Given the type of data involved there are few alternative tests: there is no theoretical justification for creating a larger number of categories, and a regression would model the relationship between individual differences and perceived emotion rather than test for differences in perceived emotion due to the individual differences. Thus, our findings may be a conservative estimate of the effects of individual differences, and should be clarified using other methods in future studies. In addition, the testing procedure uses multiple tests rather than a single model, which potentially increases the likelihood of Type I errors and is unable to reveal interactions between variables. Our analysis reduced the possible overestimation of significant effects by applying a BH correction, and the few significant effects revealed by this analysis suggests over estimation is not a problem in this case.

Second, the continuous response measure may have demand characteristics which encourage participants to model ‘emotional response’ using the only information available to them – psychophysical cues - rather than the contextual information which accompanies everyday musical encounters, such as lyrics, visual information, or the function of the music in a particular context. Rather than view this as an (undesirable) experimental artifact, this mode of listening is arguably akin to that associated with ‘autonomous’ music (Clarke, 2005). Further evidence for the validity of this behavioral approach comes from physiological data consistent with perceived (Coutinho and Cangelosi, 2009) and felt (Coutinho and Cangelosi, 2011) emotion.

Third, while the dimensional model adopted here has many advantages, both dimensional and discrete models have difficulty capturing mixed emotions of simultaneously positive and negative valence (Larsen and McGraw, 2011). Future research would need to explore the extent to which individual differences impinge on judgments of mixed emotions.

Furthermore, the music and speech stimuli did not track identical paths through the 2DES (something that would be virtually impossible to achieve with naturalistic samples), and the negative-valence, negative-arousal quadrant was absent from ratings of perceived emotion in the music stimuli and present slightly more in the speech stimuli. This imbalance in the representation of the 2DES by the music versus speech stimuli means that this study cannot offer comment on any differences (or similarities) in the impact of individual differences on perceived negative-valence, negative-arousal emotion perceived in music compared to speech. However, it does not change the overall observations which are based on a wider coverage of the 2DES.

This study captures, for the first time, the dynamic aspect of emotional experience with two types of auditory phenomena, using continuous measurement. It suggests that moment-by-moment judgments of emotion in speech prosody are unaffected by individual differences of personality, musical training, emotional intelligence and gender, but may be influenced by age, and that judgments of emotion in (novel but stylistically familiar) music are unaffected by gender and emotional intelligence and minimally influenced by personality, musical expertise and age. This is congruent with hypotheses regarding shared affective processing of auditory stimuli, but also suggests that individual differences may be more important in the context of an aesthetic realm such as music. This research also offers a new method for analyzing second-by-second emotional experiences and highlights important conceptual differences between discrete and continuous measures of emotion which future research should explore. Understanding how moment-by-moment perceptions of emotion expressed by music and speech prosody are influenced by differences between individuals will contribute to forming more nuanced models of emotional experience.

## Ethics Statement

This study was carried out in accordance with the recommendations of the University of Sheffield Ethics Policy Governing Research Involving Human Participants, Personal Data and Human Tissue, University Research Ethics Committee. The protocol was approved by the Music Department Research Ethics review panel. All subjects gave written informed consent in accordance with the Declaration of Helsinki.

## Author Contributions

ND and EC contributed to the conception and design of the study. EC performed the data collection. JV and GE-P performed the functional ANOVA tests. ND and EC wrote the first draft of the manuscript. All authors contributed to the manuscript revision, read and approved the submitted version.

## Conflict of Interest Statement

The authors declare that the research was conducted in the absence of any commercial or financial relationships that could be construed as a potential conflict of interest.
