# Supplementary material for: Do Individual Differences Influence Moment-by-Moment Reports of Emotion Perceived in Music and Speech Prosody?
Source: Front Behav Neurosci. 2018 Aug 27;12:184. doi: 10.3389/fnbeh.2018.00184 (PMC6119718; doi:10.3389/fnbeh.2018.00184)
Supplement: Supplementary file 1 [file Data_Sheet_1.docx]

Appendix

Plots showing ratings of arousal and valence for speech samples and music pieces where there are significant differences between groups.

Figure A Plot showing ratings of arousal and valence of Piece 5, grouped by high and low Emotional Stability (‘ES’).

Figure B Plot showing the ratings of arousal and valence of Piece 1, grouped by high and low Agreeableness (‘Agree’).

Figure C Plot showing the ratings of arousal and valence of Piece 2 and Piece 6, grouped by high and low musical training (‘Training’ vs ‘No training’).

Figure D Plot showing the ratings of arousal and valence of Piece 3 and Piece 7, grouped by age (‘y.o.’ = ‘years old’).

Figure E Plot showing the ratings of arousal and valence of Piece 4, grouped by age (‘y.o.’ = ‘years old’).

Figure F Plot showing the ratings of arousal and valence of Samples 5 and 8, grouped by age (‘y.o.’ = ‘years old’).

Figure G Plot showing the ratings of arousal and valence of Piece 8, grouped by gender.
